# New Insights into the Diversity of Marine Picoeukaryotes

**DOI:** 10.1371/journal.pone.0007143

**Published:** 2009-09-29

**Authors:** Fabrice Not, Javier del Campo, Vanessa Balagué, Colomban de Vargas, Ramon Massana

**Affiliations:** 1 Institut de Ciències del Mar, CSIC, Barcelona, Spain; 2 Station Biologique de Roscoff, UMR7144, Evolution du Plancton et PaléoOcéans (EPPO), Centre National de la Recherche Scientifique (CNRS) et Université Pierre et Marie Curie (UPMC), Place George Teissier, Roscoff, France; University of Alabama, United States of America

## Abstract

Over the last decade, culture-independent surveys of marine picoeukaryotic diversity based on 18S ribosomal DNA clone libraries have unveiled numerous sequences of novel high-rank taxa. This newfound diversity has significantly altered our understanding of marine microbial food webs and the evolution of eukaryotes. However, the current picture of marine eukaryotic biodiversity may be significantly skewed by PCR amplification biases, occurrence of rDNA genes in multiple copies within a single cell, and the capacity of DNA to persist as extracellular material. In this study we performed an analysis of the metagenomic dataset from the *Global Ocean Survey* (GOS) expedition, seeking eukaryotic ribosomal signatures. This PCR-free approach revealed similar phylogenetic patterns to clone library surveys, suggesting that PCR steps do not impose major biases in the exploration of environmental DNA. The different cell size fractions within the GOS dataset, however, displayed a distinct picture. High protistan diversity in the <0.8 µm size fraction, in particular sequences from radiolarians and ciliates (and their absence in the 0.8–3 µm fraction), suggest that most of the DNA in this fraction comes from extracellular material from larger cells. In addition, we compared the phylogenetic patterns from rDNA and reverse transcribed rRNA 18S clone libraries from the same sample harvested in the Mediterranean Sea. The libraries revealed major differences, with taxa such as pelagophytes or picobiliphytes only detected in the 18S rRNA library. MAST (Marine Stramenopiles) appeared as potentially prominent grazers and we observed a significant decrease in the contribution of alveolate and radiolarian sequences, which overwhelmingly dominated rDNA libraries. The rRNA approach appears to be less affected by taxon-specific rDNA copy number and likely better depicts the biogeochemical significance of marine protists.

## Introduction

In the last decade, 18S rDNA clone libraries have been considered as the gold standard approach for conducting molecular surveys of marine protist diversity in the environment [1], [2]. These investigations, almost exclusively performed on the picoplanktonic size fraction (0.2–3 µm), have unveiled high rank taxa such as the so-called MALV (marine alveolates, [3]), MAST (marine stramenopiles, [4]), and picobiliphytes [5], many of which have become cornerstone taxa for microbial ecologists. Diversity surveys of picoplanktonic protists in different marine regions have generated broadly similar patterns [2], [6], with dominance of non-photosynthetic groups, including tiny parasites [7] and grazers [8]. In contrast, epifluorescence microscopy typically reveals a dominance of photosynthetic or mixotrophic cells over heterotrophic cells (ca 80% vs 20%, respectively) in the oceans [9]. This suggests that 18S rDNA clone libraries may give a significantly biased view of diversity. Several technical limitations inherent to culture-independent explorations of microbial diversity have been highlighted [10], . Among these, biases during DNA extraction and PCR amplification steps [12], primer selectivity, multiple rDNA gene copy number [13], and the existence of pseudogenes [14] or extracellular DNA [15], are particularly relevant.

Alternative approaches focused on photosynthetic protists have recently been developed to overcome the apparent bias towards heterotrophic cells. These include the construction of clone libraries from flow cytometry sorted populations [16], studies specifically targeting plastid genes [17], and the use of taxon-specific primers [18]. However, PCR biases, rDNA copy number, and extracellular DNA remain as potentially problematic issues with these approaches. A promising alternative which does not require PCR steps is the metagenomic approach, based on direct cloning and shotgun sequencing of environmental DNA.

This strategy was recently used to study prokaryotic life on a worldwide scale (Sorcerer, Global Ocean Survey expedition, [19]). Studies that compared metagenomic and 16S rDNA PCR-based clone libraries demonstrated that these two approaches were complementary for bacterial community analysis [20], [21]. With respect to eukaryotic microbes, phylogenetic information present in metagenomic libraries has thus far received very little attention [22]. Another perspective to investigate microbial diversity is to target directly the 18S rRNA (i.e. the ribosomes themselves) as a proxy for both diversity and metabolic activity of cells [23], and to avoid the problems induced by differences in rDNA copy number and the perturbation from dissolved DNA. This approach has been proven to be effective on prokaryotic communities [11], [24], [25], but to date has only been applied on protist communities in an oxygen depleted environment [26].

In the present study we performed an in-depth analysis of the metagenomic dataset from the GOS expedition, seeking eukaryotic signatures through the presence of 18S rDNA genes. We also compared the protist diversity assessed by 18S rDNA libraries prepared from both environmental DNA and RNA extracted from the same water sample collected in the Mediterranean Sea. We show that overall the PCR induced biases do not appear to impact significantly diversity surveys. Rather we argue that rDNA copy number and extracellular DNA (partially by-product of the size fractionation) are major issues that introduce biases in current studies of protist diversity. Environmental 18S rRNA clone libraries appear to represent a promising means to minimize these biases and thereby offer new perspectives in the study of the diversity and function of marine protist.

## Results

### Taxonomic composition in 18S rDNA clone libraries versus the metagenomic dataset

**Figure 1:**
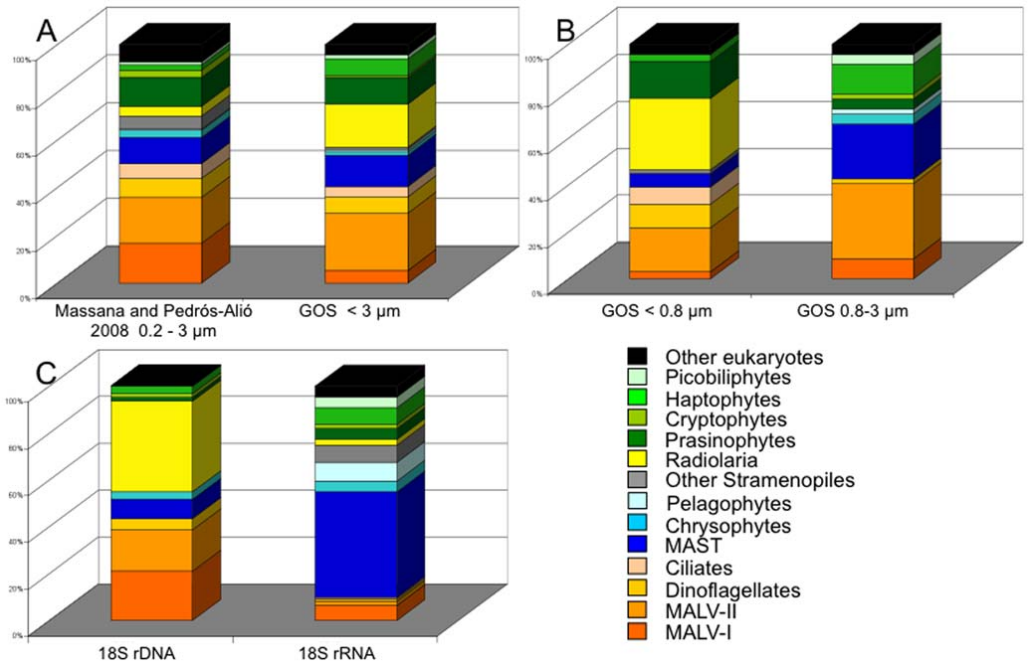
Relative contribution of different taxonomic groups from 18S rDNA sequences obtained from the picoplankton fraction of marine samples. A. Comparison of data obtained through PCR-based clone libraries as presented in [2] *versus* the metagenomic data retrieved from < 3 µm size fraction of the GOS dataset. B. Detail of the metagenomic GOS dataset obtained from two different size fractions <0.8 µm and 0.8 to 3 µm. C. Comparison of clone libraries performed on the same sample from the Mediterranean Sea (0.6 to 3 µm size fraction) after DNA extraction (62 sequences) and RNA extraction (111 sequences). Actual numbers of sequences affiliated to each taxonomic group used to prepare these graphs are shown in Table S1.

Taxonomic affiliation of sequences retrieved from PCR amplified 18S rDNA clones libraries performed on the picoplankton size fraction (0.2 to 3 µm) of samples collected in the photic zone around the globe [2] was compared to that of 18S rDNA sequences found in the <3 µm size fraction of the GOS metagenomic dataset (Figure 1A). Despite the large differences in the number of sequences analyzed for both datasets, random sub-sampling of the larger dataset demonstrated that the range of expected averaged distributions on a smaller number of sequences matched closely to the distribution observed (Figure S1). This shows that looking at a limited number of sequences does not affect the diversity observed at the taxonomic level we considered. The clonal representation of the different taxonomic groups in both datasets was significantly correlated (slope 0.78; R^2^ = 0.39; p = 0.0165), indicating that both integrated datasets yielded comparable results.

In the clone libraries, out of the 2175 sequences reviewed by Massana and Pedrós-Alió, alveolates dominated the assemblages with 50.3% of the sequences retrieved (most of which were MALV, marine alveolates: 19.2% MALV-II and 16.7% MALV-I). The second most represented taxon was the stramenopiles, accounting for 20% of the eukaryotic sequences (of which 10.9% were MAST, marine stramenopiles). Prasinophytes and radiolarians accounted for 12.1% and 4.1% of the sequences, respectively. Cryptophytes, haptophytes and picobiliphytes represented 2.9%, 2.4%, and 1.1% of the sequences. The category “other”, accounting for 7.2% of the sequences, was mainly composed of cercozoans, choanoflagellates and unassigned alveolates. Out of 116 sequences extracted from the GOS metagenomic dataset, the most represented groups were the alveolates (40.5%, of which 24.1% belonged to MALV-II and 5.2% to MALV-I), radiolarians (18.1%), stramenopiles (16.4%, including 12.9% of MAST), prasinophytes (11.2%), and haptophytes (6.9%). Sequences affiliated to the picobiliphytes accounted for 1.7% of total sequences.

The 18S rDNA sequences retrieved from the GOS dataset had an averaged similarity of 96.0% with sequences deposited in the GenBank database. The most dissimilar sequences affiliated with marine alveolates (e.g. 80.6% similarity), whereas some were identical to GenBank entries (mostly prasinophytes and the haptophyte *Chrysochromulina*) and many were >99% similar to GenBank sequences. Closest matches for most GOS sequences corresponded to environmental clones obtained from PCR-based studies (Tables S4 and S5)

### Taxonomic analysis of distinct size fractions among the metagenomic dataset

Of the 116 18S rDNA sequences identified in the metagenomic dataset from the GOS expedition, 69 derived from the <0.8 µm fraction and 47 from the 0.8–3 µm size fraction. Clearly, both size fractions were capturing a distinct fraction of picoeukaryotic diversity (Figure 1B), and the percentage of taxonomic groups observed in the two size fractions did not correlate at all (slope 0.18; R^2^ = 0.03; p = 0.5523). Considering the smaller size fraction (<0.8 µm), radiolarians contributed 30.4% and stramenopiles 7.2% of the sequences (with 5.8% MAST). The overall contribution of alveolates was 41.9% of the sequences, including 18.8% of MALV-II, 10.1% of dinoflagellates and 7.2% of ciliates. Prasinophytes contributed 15.9% and haptophytes 2.9%. No picobiliphyte sequences were detected. In the larger size fraction (0.8–3 µm) the overall contribution of alveolates remained similar, but there was an increase of MALV-II (31.9% of sequences) and a decrease of dinoflagellates (2.1%) and ciliates (not detected). The contribution of stramenopiles increased drastically to 29.8% (of which 23.4% were MAST) while not a single radiolarian sequence was identified. Prasinophytes decreased to 4.3%, whereas the contributions of haptophytes, chrysophytes, and picobiliphytes increased to 12.8%, 4.3%, and 4.3%, respectively.

### 18S rDNA clones libraries prepared from DNA and RNA extracts

18S rDNA environmental clone libraries were constructed from DNA and RNA extracts (followed by a reverse transcription) obtained from the same seawater sample (Figure 1C). Considering the limited number of clones sequenced and previous knowledge for marine samples, the libraries were explored in numbers far from saturation. Nevertheless, obvious patterns could be distinguished and the distribution of diversity observed for the 18S rRNA library is well outside the range of expected values for 18S rDNA libraries. Again, there was no correlation among the clonal percentage of taxonomic groups in the two libraries (slope -0.02; R^2^ = 0.00; p = 0.9539). Among the 62 sequences from the DNA based library, 43.5% affiliated to alveolates, 38.7% to radiolarians, and 11.3% to stramenopiles. Most alveolate sequences affiliated with MALV-I (21.0%) or MALV-II (17.7%). Most of the stramenopiles belonged to MAST (i.e. 8.1% of the sequences). Chrysophytes, haptophytes, prasinophytes, and cryptophytes were detected but with a low clonal representation. In the rRNA based library, the diversity observed for the 111 sequences analyzed was drastically different. The contribution of alveolates decreased to 9.9% and the contribution of stramenopiles increased to 64.8% including 45.0% MAST. The contribution of sequences affiliated to haptophytes and prasinophytes increased to 7.2% and 4.5%, respectively. In contrast, the contribution of radiolarians sharply decreased down to 2.7%. The pelagophytes and picobiliphytes, which were not detected in the DNA survey, contributed 8.1% and 4.5% of sequences in the RNA survey, respectively. Also only detected in the RNA-based library, dictyochophytes made up half of the “other stramenopiles” category and *Telonemia* the major fraction of the “other eukaryotes” (data not shown).

In each library, Operational Taxonomic Units (OTUs) were defined using a 99% identity threshold (Table 1). Of the 62 and 111 sequences from the DNA and RNA based libraries, 34 and 52 OTUs were identified, respectively. Only 2 OTUs were present in both libraries, one affiliated to MALV-I, and the other to MAST-4. Using a 98% identity threshold, 29 and 46 OTUs were identified for the DNA and RNA based libraries respectively, but only one additional OTU (belonging to chrysophytes) was common to the two libraries. Statistical comparisons performed with LIBSHUFF found a significant difference between the two libraries (p<0.001).

**Table 1:** Number of sequences and OTUs (Operational Taxonomic Units) defined at 99% identity threshold in different taxonomic groups from both DNA- and RNA-based libraries.

	DNA		RNA	
	# seq.	OTU 99%	# seq.	OTU 99%
MALV-I	13	8	7	2
MALV-II	11	8	2	2
Dinoflagellates	3	2	1	1
Ciliates	0	0	1	1
MAST	5	3	50	20
Chrysophytes	2	1	5	2
Pelagophytes	0	0	9	1
Other Stramenopiles	0	0	8	6
Radiolarians	24	8	3	2
Prasinophytes	1	1	5	3
Cryptophytes	1	1	2	1
Haptophytes	2	2	8	6
Picobiliphytes	0	0	5	1
Telonema	0	0	3	2
Other	0	0	2	2
**TOTAL**	**62**	**34**	**111**	**52**
**Ratio OTUs / # seq.**	**0.55**		**0.47**	

## Discussion

### 18S rDNA clone libraries and metagenomic surveys give similar diversity patterns

Our analyses of the 18S rDNA sequences retrieved from the metagenomic dataset from the GOS expedition did not reveal substantial differences as compared to the PCR-based environmental clone libraries (Figure 1A). Both datasets were obtained from a similar size fraction (<3 µm) and correspond to compilations of sequences from various sampling locations and thus represent a reasonable integration of the photic layer in the marine environment. Eukaryotic microbial diversity assessed by means of environmental clone libraries of the 18S rDNA gene has been reported from a variety of ecosystems over the last decade [2], [6]. This approach has led to the discovery of eukaryotic taxa such as the MALV and MAST groups that often dominate the community in terms of clonal abundance. Among the technical issues usually invoked to lead to biases in 18S rDNA clone libraries there is the PCR step before the cloning procedure [10], [12]. Metagenomic approaches directly clone and shotgun sequence the DNA from a given sample, without prior PCR. The similarity in diversity patterns between the PCR cloning and metagenomic approaches suggests little impact of the PCR step on the outcome of clone libraries in terms of sequence diversity and relative contribution of specific taxa. Our observation is consistent with similar studies on 16S rDNA bacterial diversity that did not find significant differences at high phylogenetic levels between metagenomic and PCR-based libraries [20].

### Analysis of GOS size fractions refines our view of actual community composition

Separate analysis of the two size fractions from the GOS dataset revealed clear differences in terms of taxonomic composition (Figure 1B). As the smallest eukaryotic organism known so far has a cell diameter of 0.8 µm [27], some of the 18S rDNA signatures observed in the <0.8 µm fraction might indeed derive from very small eukaryotes (like the prasinophytes that appeared mostly in this small fraction, Table S4), but many sequences most likely derive from cell debris or extracellular DNA from larger cells. This is likely the case for radiolarians, dinoflagellates, and ciliates, groups known to contain relatively large nano- and microplanktonic cells, and for which sequences were prominent in the <0.8 µm fraction and nearly absent from the 0.8–3 µm fraction. Among these groups, the radiolarians were the most intriguing, since these relatively large exoskeleton bearing protists typically represent a significant fraction of 18S rDNA sequences in diversity surveys of marine picoeukaryotes (Figure 1A). These radiolarian sequences appear highly diverse [28], and most likely derived from larger organisms for which molecular data are not yet available, highlighting the extent of both the unknown diversity in this taxonomic group and filtration artifacts which affect environmental surveys of the smallest size fractions. As suggested in a recent study that investigated the eukaryotic diversity of the <0.8 µm size fraction in a subset of the GOS dataset (i.e. Sargasso Sea samples) [22], future environmental surveys should target the 0.8–3 µm fraction, which may actually better represent the picoeukaryote diversity.

Several studies have proved the occurrence of extracellular DNA (particulate or dissolved) in water or sediments [29]–[31]. Among this DNA pool, a substantial portion contains high molecular weight molecules and is thought to be derived from eukaryotic organisms [29]. This extracellular DNA is prone to PCR amplification, and genes such as the one coding for the rbcL enzyme have been successfully amplified from particle-free water samples [15]. It is very likely that a fraction of the extracellular DNA is retained onto 0.2 µm filters, through collection of aggregates or molecular adsorption. Consequently, we believe that it is important to consider the interference of extracellular DNA when assessing the diversity of eukaryotic microbes in ecological perspectives.

### The RNA approach gives complementary perspectives on marine protist diversity

Diversity assessed by means of libraries prepared after reverse transcription of extracted RNA led to a drastically different view of the community as compared to the classical DNA-based approach (Figure 1C). It is generally recognised that 18S rDNA diversity surveys are not quantitative with respect to cell abundance [32], [33]. Besides PCR biases, the contributions of specific taxa are related to the number of rDNA copies within cells of the taxa. Although rDNA copy number is usually assumed to be correlated with cell size [13], [34], for a limited size range (e.g. picoeukaryotes) this number can vary significantly depending on phylogenetic affiliation and is also suspected to be influenced by life strategies of cells (e.g. parasitic, heterotrophic, autotrophic) [2], [35]. The effect of taxon-specific rDNA copy number is avoided when analysing extracted RNA. Moreover, extracellular RNA is much less stable than DNA, minimising the problem of amplification from extracellular material. Ribosome content within a single cell is commonly viewed as a proxy of cellular activity status [23], [36]. Therefore, 18S rRNA libraries are intentionally skewed to give insights on both diversity and taxon specific activity within protist assemblages [26]. As a flip side effect we might have expected an over representation of the most active taxa. However, both DNA-based and RNA-based libraries contained a high diversity, with comparable ratios of OTUs/number of sequences (Table 1). We found very little overlap in the sequences retrieved in the DNA and RNA libraries. At the 98% identity threshold, only 3 OTUs (ca. 4%) were detected in both libraries, which is rather low compared to the 27% observed in a similar study on anoxic waters [26]. This discrepancy might be explained by a lower sequencing effort done here but also by the selective nature of anoxic waters that might impose stronger constraints on the communities compared with open ocean conditions, implying a lower diversity and therefore a higher overlap between rDNA and rRNA libraries.

The diversity observed by both approaches is clearly not distributed within the same high level taxa, paralleling observations made on prokaryotes or on eukaryotes in an extreme environment [11], [24]–[26]. Some photosynthetic groups such as pelagophytes and picobiliphytes were not detected in the 18S rDNA based library, whereas they contributed notably to the 18S rRNA library (Figure 1C). The relative contribution of other photosynthetic groups such as the prasinophytes and the haptophytes was also higher in the rRNA library. This might reflect a relatively higher metabolic activity in these photosynthetic taxa at the time of sampling, or may indicate that they have fewer rDNA copies (e.g. *Pelagomonas*, [13]), so they could be diluted in the environmental DNA surveys by cells with a higher rDNA copy number (e.g. alveolates). Among prasinophytes, cells belonging to the genus *Micromonas* were identified as being the most active (Table S3), confirming previous studies showing the significance of this genus in coastal ecosystems [37]. Regarding heterotrophic protists, sequences belonging to MAST-3, -4 and -7 appeared as prominent grazers (Table S3), which together with the widespread distribution of these taxa suggest they might actually be the major protistan predators in the oceans [8]. Finally, the most pronounced divergence between both libraries was the contribution of alveolates and radiolarians, which overwhelmingly dominated DNA-based diversity surveys [2]. This perhaps reflects the high 18S rDNA gene diversity and high copy number matching the parasitic life strategy of MALV [7], [38] and further supports the putative presence of extracellular radiolarian 18S rDNA in seawater.

### Conclusions

Size fractionation, metagenomics, and 18S rRNA libraries bring new perspectives for the understanding of marine picoeukaryotic diversity. In particular, rRNA libraries reduce significantly two of the major biases of rDNA diversity surveys, the rDNA copy number and the occurrence of extracellular DNA, but are in turn skewed towards the active part of the communities. Considering the relative ease of handling ribosomal RNA molecules, extended diversity surveys based on environmental rRNA will undoubtedly provide insights into the ecology of uncultured species. Associated with stronger depth of sequencing (e.g. 454 [39]), this approach will probably help to achieve a nearly exhaustive view of protist diversity and to better appreciate the contribution and function of specific organisms in the microbial food web.

## Materials and Methods

### Mining the GOS dataset using CAMERA

The Global Ocean Survey (GOS) covered a variety of oceanic regions from Nova Scotia to South Africa across the Caribbean, the Panama Channel, the Pacific and the Indian Ocean [19] and data is accessible through the CAMERA database [40]. For the purpose of our analysis, and to compare waters of similar characteristics, only samples from offshore and coastal photic zones were used, whereas samples from environments such as hypersaline lagoons or mangroves were discarded. Seventy two sampling sites, representing a sequencing effort of 14000 Mb, were analyzed for the <0.8 µm fraction, whereas only 8 sampling sites (850 Mb) were analyzed for the 0.8–3 µm fraction. This demonstrates the primary focus on prokaryotes of the GOS expedition. The fraction <3 µm recorded in our analysis corresponds to the sum of data retrieved from the two size fractions. We searched for 18S rDNA genes using the eukaryotic specific primers EukA and EukB [41], 528f [42], 336f and 1209f [43] as *in silico* probes. Sequences were then assigned to specific taxonomic groups after the results of BLAST searches [44]. Chimeras were detected by doing BLAST with different regions of the sequence. Metazoans, marine euryarchaeote group II sequences (obtained with EukA primer), and short (<100 bp) sequences were discarded. We ended up with a total of 116 eukaryotic sequences from this metagenomic survey, with 69 and 47 sequences in <0.8 µm and 0.8–3 µm size fractions, respectively.

### Sampling procedures for the DNA vs RNA clone libraries

Seawater samples were harvested on November, 15^th^ 2007 in the Mediterranean Sea off Villefranche sur Mer (France). Water was collected with a 12L Niskin bottle deployed successively at 40, 60, 80, 100, 120, and 140 meter depths. After a pre-filtration through a 1000 µm mesh, equal volumes of water from each depth were mixed together in order to obtain an integration of the communities throughout the water column. Then water was gently sieved through 63 µm and 20 µm meshes and filtered through a 3 µm pore size 47 mm diameter polycarbonate filter. For DNA and RNA libraries, around 4 liters of the fraction below 3 µm were filtered onto 0.6 µm pore size 47 mm diameter polycarbonate filters at a rate of 90 ml min^−1^. Finally the filters were flash frozen in liquid nitrogen and stored at −80°C until further analysis.

### Nucleic acid extractions and clone library construction

For DNA extraction, the filter was cut in small pieces with a sterile razor blade and placed in a 2 ml microcentrifuge tube. Liquid nitrogen was added to the tube and the frozen sample material was disrupted manually with a disposable pellet pestle (Fisher Bioblock), repeating this step four more times. This disruption procedure was followed by DNA extraction with a DNeasy Plant Mini kit (Qiagen) following the manufacturer's recommendations. DNA extracts were stored at −80°C until analysis. For RNA extraction, filters were immersed in RLT buffer (from a Quiagen RNeasy kit) mixed with an equal amount of 0.1 and 0.5 µm glass beads and subsequently vortexed. Then the RNeasy kit instructions for Plants and Fungi were followed. Quantification of extracted nucleic acids was performed with the Qubit Quantitation platform (Invitrogen). Prior to reverse transcription, a DNase digestion step was performed with DNaseI (Roche Diagnostic) and efficient digestion was controlled by gel electrophoresis. Reverse transcription was performed on pure RNA using the SuperScript II kit (Invitrogen) according to the manufacturer's instructions. The eukaryotic 18S specific EUKB primer [41] was used for the reverse transcription.

Both 18S rDNA genes and 18S ribosomal cDNA were PCR amplified using the same set of primers, 528f [42] and EUKB [41]. Approximately 10 ng of DNA were used as a template in a 50 µl PCR mixture containing 200 µM of each dNTP, 1.5 mM MgCl_2_, 0.5 µM of each primer and 1.25 units of *Taq* DNA polymerase (Promega) with the PCR buffer supplied with the enzyme. Reactions were carried out in a thermocycler with the following cycle: an initial denaturing step at 94°C for 3 min, 35 cycles of denaturing at 94°C for 45 s, annealing at 55°C for 1 min and extension at 72°C for 3 min, and a final extension step at 72°C for 10 min. In order to check the quality of the RNA extraction, we used the RNA extract digested by DNase as a PCR template. Negative results confirmed the lack of remnant DNA after digestion which could have interfered with the results obtained for the cDNA libraries. PCR products were used for clone library construction. In both cases, three separate fresh PCR products (50 µl) were pooled and cleaned with the Qiagen PCR Purification kit and cloned using the TOPO-TA cloning kit (Invitrogen). Putative positive clones were checked by PCR amplification using the same primer set. PCR reactions showing the right insert size were purified and sequenced with the 528f primer on an ABI Prism 3100 sequencer (Applied Biosystems) at the Station Biologique de Roscoff sequencing facility.

Taxonomic affiliation of the 18S rDNA sequences obtained in this study (between 800 and 950 bp length) and putative chimeras were identified by using BLAST as explained before (data shown in Tables S2 and S3). Among the 113 cDNA clones sequenced 2 were chimeras leaving 111 sequences for further analysis. Sixty seven rDNA clones were sequenced, 2 chimeras were identified, and 3 metazoan sequences (Appendicularia and copepods) were discarded, leaving 62 sequences for further analysis. Operational Taxonomic Units (OTU) at 99% identity threshold were identified and compared among libraries using the DOTUR and SONS programs [45], [46]. Statistical comparisons of the two libraries were performed with the webLIBSHUFF tool [47]. Sequences have been deposited in GenBank under accession numbers GQ344621 to GQ344796.

### Statistical analysis

Considering the small number of sequences retrieved from our analysis, we wanted to make sure that comparisons between datasets were meaningful. Using R software we calculated the expected distribution of sequences from small size samples compared to a larger reference dataset. The random sub-sampling procedure of 62 and 47 sequences was replicated 1000 times from the Massana and Pedrós-Alió (2008) dataset (2175 sequences) and the GOS dataset (116 sequences), respectively. Standard deviations were calculated for each taxonomic group considered and comparisons between observed and expected datasets were plotted (Figure S1).

Correlations were performed with the statistical package JMP 5.0.1a to evaluate the degree of divergence between paired datasets and estimate the impact of PCR approaches (Figure 1A), size fractionation (Figure 1B), and 18S rDNA versus 18S rRNA clones libraries (Figure 1C), on environmental diversity surveys.

## Supporting Information

Figure S1 Taxonomic distribution of observed diversity compared to expected distribution in a sample of smaller size. A) Histogram showing the observed distribution of sequences in the Massana and Pedrós-Alió 2008 dataset (Black) and the average and standard deviation of expected distribution after random sub-sampling of 62 sequences, replicated 1000 times (Red). B) Histogram showing the observed distribution of sequences in the GOS < 3µm dataset (Black) and the average and standard deviation of expected distribution after random sub-sampling of 47 sequences, replicated 1000 times (Red).
(3.02 MB TIF)

Table S1 Number of sequences for each taxonomic group found in the analyzed dataset
(0.05 MB DOC)

Table S2 List of closest blast results for the RNA based clone library
(0.26 MB DOC)

Table S3 List of closest blast results for the DNA based clone library
(0.16 MB DOC)

Table S4 Closest blast hits on sequences retrieved from the GOS < 0.8µm dataset
(0.10 MB DOC)

Table S5 Closest blast hits on sequences retrieved from the GOS 0.8 - 3 µm dataset
(0.08 MB DOC)

## References

[1] Epstein S, Lopez-Garcia P (2008). “Missing” protists: a molecular prospective.. Biodiversity and Conservation.

[2] Massana R, Pedros Alió C (2008). Unveiling new microbial eukaryotes in the surface ocean.. Current Opinion in Microbiology.

[3] López García P, Rodriguez Valera F, Pedros Alió C, Moreira D (2001). Unexpected diversity of small eukaryotes in deep-sea Antarctic plankton.. Nature.

[4] Massana R, Castresana J, Balague V, Guillou L, Romari K (2004). Phylogenetic and ecological analysis of novel marine stramenopiles.. Applied and Environmental Microbiology.

[5] Not F, Valentin K, Romari K, Lovejoy C, Massana R (2007). Picobiliphytes: A marine picoplanktonic algal group with unknown affinities to other eukaryotes Science.

[6] Worden AZ, Not F, Kirchman DL (2008). Ecology and diversity of picoeukaryotes.. Microbial Ecology of the Ocean. 2nd edition ed.

[7] Guillou L, Viprey M, Chambouvet A, Welsh RM, Kirkham AR (2008). Widespread occurrence and genetic diversity of marine parasitoids belonging to *Syndiniales* (Alveolata).. Environmental Microbiology.

[8] Massana R, Terrado R, Forn I, Lovejoy C, Pedrós-Alió C (2006). Distribution and abundance of uncultured heterotrophic flagellates in the world oceans.. Environmental Microbiology.

[9] Jürgens K, Massana R, Kirchman DL (2008). Protist grazing on marine bacterioplankton.. Microbial Ecology of the Oceans. 2nd edition ed.

[10] Wintzingerode Fv, Göbel UB, Stackebrandt E (1997). Determination of microbial diversity in environmental samples: pitfalls of PCR-based rRNA analysis.. Fems Microbiology Review.

[11] Moeseneder MM, Arrieta JM, Herndl GJ (2005). A comparison of DNA- and RNA-based clone libraries from the same marine bacterioplankton community.. Fems Microbiology Ecology.

[12] Suzuki MT, Giovannoni SJ (1996). Bias Caused by Template reannealing in the Amplification of Mixtures of 16S rRNA Genes by PCR.. Applied and Environmental Microbiology.

[13] Zhu F, Massana R, Not F, Marie D, Vaulot D (2005). Mapping of picoeucaryotes in marine ecosystems with quantitative PCR of the 18S rRNA gene.. FEMS Microbial Ecology.

[14] Thornhill DJ, LaJeunesse TC, Santos SR (2007). Measuring rDNA diversity in eukaryotic microbial systems: how intragenomic variation, pseudogenes, and PCR artifacts confound biodiversity estimates.. Molecular Ecology.

[15] Paul JH, Cazares L, Thurmond J (1990). Amplification of the rbcL Gene from Dissolved and Particulate DNA from Aquatic Environments.. Applied and Environmental Microbiology.

[16] Shi X, Marie D, Vaulot D (submitted) Novel photosynthetic lineages uncovered in the South East Pacific Ocean from flow cytometry sorted picoeukaryote populations..

[17] Fuller NJ, Campbell C, Allen DJ, Pitt FD, Zwirglmaier K (2006). Analysis of photosynthetic picoeukaryote diversity at open ocean sites in the Arabian Sea using a PCR primer biased towards marine algal plastids.. Aquatic Microbial Ecology.

[18] Liu H, Probert I, Uitz J, Claustre H, Aris-Brossou S (2009). Extreme diversity in noncalcifying haptophytesexplains a major pigment paradox in open oceans.. Proceedings of the National Academy of Sciences in press.

[19] Rusch DB, Halpern AL, Sutton G, Heidelberg KB, Williamson S (2007). The Sorcerer II Global Ocean Sampling expedition: Northwest Atlantic through eastern tropical Pacific.. PloS Biology.

[20] Cottrell MT, Waidner LA, Yu L, Kirchman DL (2005). Bacterial diversity of metagenomic and PCR libraries from the Delaware River.. Environmental Microbiology.

[21] Liles MR, Manske BF, Bintrim SB, Handelsman J, Goodman RM (2003). A census of rRNA genes and linked genomic sequences within a soil metagenomic library.. Applied and Environmental Microbiology.

[22] Piganeau G, Desdevises Y, Derelle E, Moreau H (2008). Picoeukaryotic sequences in the Sargasso Sea metagenome.. Genome Biology.

[23] Poulsen LK, Ballard G, Stahl DA (1993). Use of rRNA Fluorescence In Situ Hybridization for Measuring the Activity of Single Cells in Young and Established Biofilms.. Applied and Environmental Microbiology.

[24] Gentille G, Giuliano L, D'Auria G, Smedile F, Azzaro M (2006). Study of bacterial communities in Antarctic coastal waters by a combination of 16S rRNA and 16S rDNA sequencing.. Environmental Microbiology.

[25] Mills HJ, Martinez RJ, Story S, Sobecky PA (2005). Characterization of Microbial Community Structure in Gulf of Mexico Gas Hydrates: Comparative Analysis of DNA- and RNA-Derived Clone Libraries.. Applied and Environmental Microbiology.

[26] Stoeck T, Zuendorf A, Breiner H-W, Behnke A (2007). A molecular approach to identify active microbes in environmental eukaryote clone libraries.. Microbial Ecology.

[27] Courties C, Vaquer A, Trousselier M, Lautier J, Chrétiennot-Dinet M-J (1994). Smallest eukaryotic organism.. Nature.

[28] Not F, Gausling R, Azam F, Heidelberg JF, Worden AZ (2007). Vertical distribution of picoeukaryotic diversity in the Sargasso Sea.. Environmental Microbiology.

[29] Jiang SC, Paul JH (1995). Viral Contribution to Dissolved DNA in the Marine Environment as Determined by Differential Centrifugation and Kingdom Probing.. Applied and Environmental Microbiology.

[30] Dell'Anno A, Danovaro R (2005). Extracellular DNA Plays a Key Role in Deep-Sea Ecosystem Functioning.. Science.

[31] Vlassov VV, Laktionov PP, Rykova EY (2007). Extracellular nucleic acids.. BioEssays.

[32] Kirchman DL (2002). The ecology of Cytophaga-Flavobacteria in aquatic environments.. Fems Microbiology Ecology.

[33] Not F, Latasa M, Scharek R, Viprey M, Karleskind P (2008). Phytoplankton diversity across the Indian Ocean: A focus on the picoplanktonic size fraction.. Deep - Sea Research Part I - Oceanographic Research Papers.

[34] Godhe A, Asplund ME, Härnström K, Saravanan V, Tyagi A (2008). Quantifying diatom and dinoflagellate biomass in coastal marine seawater samples by real-time PCR.. Applied and Environmental Microbiology.

[35] Vaulot D, Romari K, Not F (2002). Are autotrophs less diverse than heterotrophs in marine picoplankton?. Trends in Microbiology.

[36] Buckley BA, Szmant AM (2004). RNA/DNA ratios as indicators of metabolic activity in four species of Caribbean reef-building corals.. Marine Ecology - Progress Series.

[37] Not F, Latasa M, Marie D, Cariou T, Vaulot D (2004). A single species, *Micromonas pusilla* (Prasinophyceae), dominates the eukaryotic picoplankton in the Western English Channel.. Applied and Environmental Microbiology.

[38] Chambouvet A, Morin P, Marie D, Guillou L (2008). Control of toxic marine dinoflagellate blooms by derial parasitic killers.. Science.

[39] Margulies M, Egholm M, Altman WE, Attiya S, Bader JS (2005). Genome sequencing in microfabricated high-density picolitre reactors.. Nature.

[40] Seshadri R, Kravitz SA, Smarr L, Gilna P, Frazier M (2007). CAMERA: A Community Resource for Metagenomics.. PloS Biology.

[41] Medlin LK, Elwood HJ, Stickel S, Sogin ML (1988). The characterization of enzymatically amplified eukaryotic 16S-like r RNA-coding regions.. Gene.

[42] Elwood HJ, Olsen GJ, Sogin ML (1985). The small-subunit ribosomal RNA gene sequences from the hypotrichous ciliates *Oxytricha nova* and *Stylonychia pustulata*.. Molecular Biology and Evolution.

[43] Giovannoni SJ, DeLong EF, Olsen GJ, Pace NR (1988). Phylogenetic group-specific oligonucleotide probes for identification of single microbial cells.. Journal of Bacteriology.

[44] Altschul StephenF, Madden ThomasL, Schäffer AlejandroA, Zhang Jinghui, Zhang Zheng, Miller Webb, Lipman DavidJ (1997). “Gapped BLAST and PSI-BLAST: a new generation of protein database search programs”, Nucleic Acids Res..

[45] Schloss PD, Handelsman J (2005). Introducing DOTUR, a Computer Program for Defining Operational Taxonomic Units and Estimating Species Richness.. Applied and Environmental Microbiology.

[46] Schloss PD, Handelsman J (2006). Introducing SONS, a Tool for Operational Taxonomic Unit-Based Comparisons of Microbial Community Memberships and Structures.. Applied and Environmental Microbiology.

[47] Singleton DR, Furlong MA, Rathbun SL, Whitman WB (2001). Quantitative Comparisons of 16S rRNA Gene Sequence Libraries from Environmental Samples.. Applied and Environmental Microbiology.

