# Supplementary figures and images for: New Insights into the Diversity of Marine Picoeukaryotes
Source: PLoS One. 2009 Sep 29;4(9):e7143. doi: 10.1371/journal.pone.0007143 (PMC2747013; doi:10.1371/journal.pone.0007143)

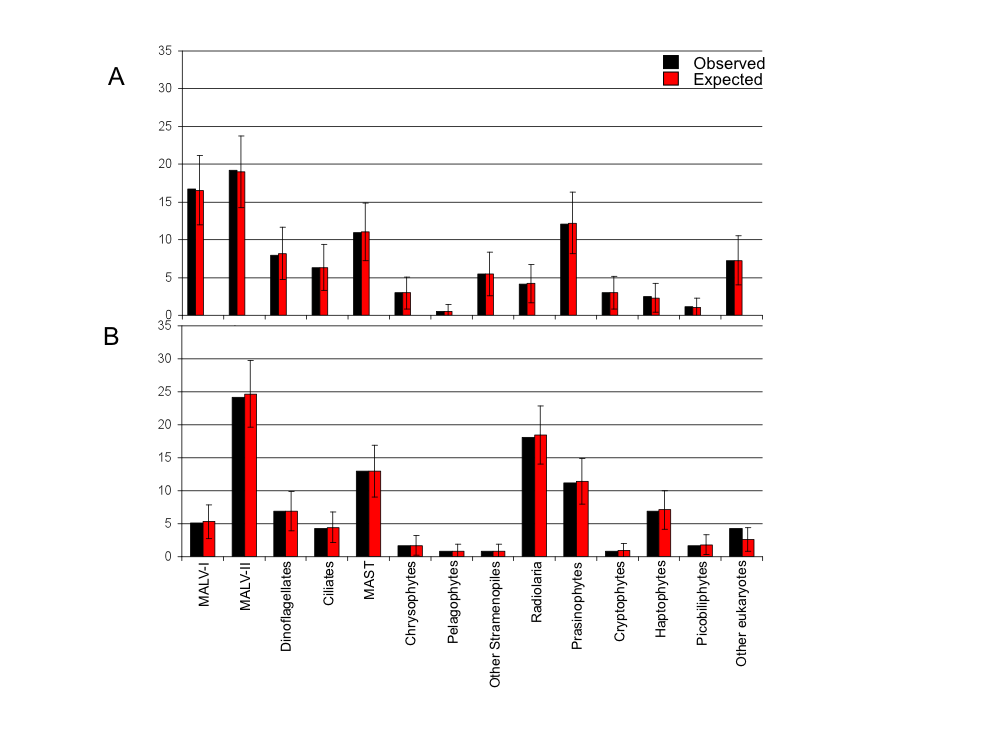

Supplement: Figure S1 — Taxonomic distribution of observed diversity compared to expected distribution in a sample of smaller size. A) Histogram showing the observed distribution of sequences in the Massana and Pedrós-Alió 2008 dataset (Black) and the average and standard deviation of expected distribution after random sub-sampling of 62 sequences, replicated 1000 times (Red). B) Histogram showing the observed distribution of sequences in the GOS < 3µm dataset (Black) and the average and standard deviation of expected distribution after random sub-sampling of 47 sequences, replicated 1000 times (Red). (3.02 MB TIF) [file pone.0007143.s001.tif]
