# Supplementary material for: New Insights into the Diversity of Marine Picoeukaryotes
Source: PLoS One. 2009 Sep 29;4(9):e7143. doi: 10.1371/journal.pone.0007143 (PMC2747013; doi:10.1371/journal.pone.0007143)
Supplement: Table S1 — Number of sequences for each taxonomic group found in the analyzed dataset (0.05 MB DOC) [file pone.0007143.s002.doc]

|  |  | Massana et al 2008 | DNA VFR | RNA VFR | GOS | GOS | GOS |
| --- | --- | --- | --- | --- | --- | --- | --- |
|  |  | 0.2 – 3 µm | 0.6 - 3 µm | 0.6 - 3 µm | 0.2 - 3 µm | 0.2 - 0.8 µm | 0.8 - 3 µm |
| Alveolata | MALV-I | 363 | 13 | 7 | 6 | 2 | 4 |
|  | MALV-II | 417 | 11 | 2 | 28 | 13 | 15 |
|  | Dinophyceae | 173 | 3 | 1 | 8 | 7 | 1 |
|  | Ciliophora | 137 | 0 | 1 | 5 | 5 |  |
| Stramenopiles | MAST | 237 | 5 | 50 | 15 | 4 | 11 |
|  | Chrysophyceae | 65 | 2 | 5 | 2 |  | 2 |
|  | Pelagophyceae | 12 | 0 | 9 | 1 |  | 1 |
|  | Other | 120 | 0 | 8 | 1 | 1 |  |
| Radiolaria |  | 90 | 24 | 3 | 21 | 21 |  |
| Prasinophyceae |  | 263 | 1 | 5 | 13 | 11 | 2 |
| Cryptophyta |  | 64 | 1 | 2 | 1 |  | 1 |
| Haptophyta |  | 53 | 2 | 8 | 8 | 2 | 6 |
| Picobiliphytes |  | 24 | 0 | 5 | 2 |  | 2 |
| Other |  | 157 | 0 | 5 | 5 | 3 | 2 |
| **TOTAL** |  | **2175** | **62** | **111** | **116** | **69** | **47** |
