# Supplementary material for: New Insights into the Diversity of Marine Picoeukaryotes
Source: PLoS One. 2009 Sep 29;4(9):e7143. doi: 10.1371/journal.pone.0007143 (PMC2747013; doi:10.1371/journal.pone.0007143)
Supplement: Table S2 — List of closest blast results for the RNA based clone library (0.26 MB DOC) [file pone.0007143.s003.doc]

| Name | read | Closest match | % | Phylogenetic group | Closest cultured match | % | Shorter seq |
| --- | --- | --- | --- | --- | --- | --- | --- |
| cDNA.21 | 839 | SSRPD64 | 100.0 | Chrysophyte | Ochromonas CCMP1278 | 94.7 |  |
| cDNA.28 | 839 | SSRPD64 | 100.0 | Chrysophyte | Ochromonas CCMP1278 | 94.7 |  |
| cDNA.74 | 915 | C3_E031 | 99.9 | Chrysophyte | Mallomonas annulata | 92.9 |  |
| cDNA.75 | 905 | SSRPD64 | 98.3 | Chrysophyte | Mallomonas annulata | 91.4 |  |
| cDNA.91 | 949 | SSRPD64 | 98.4 | Chrysophyte | Mallomonas annulata | 91.7 |  |
| cDNA.05 | 886 | TH10.53 | 93.1 | Ciliate | F.blochmanni | 90.5 |  |
| cDNA.46 | 910 | SSRPD79 | 99.3 | Cryptomonas | Chroomonas sp. M1312 | 89.0 | BL000921.41 (99,5%) |
| cDNA.50 | 839 | SIF_4C4 | 98.4 | Cryptophyte-nucleomorph | Geminigera cryophila | 93.3 |  |
| cDNA.39 | 835 | CD8.07 | 92.9 | Dictyochophyceae | Pedinellales sp. CCMP2098 | 93.0 |  |
| cDNA.43 | 897 | Pedinellales sp. CCMP2098 | 93.3 | Dictyochophyceae |  |  |  |
| cDNA.48 | 898 | Q2F10N5 | 99.1 | Dictyochophyceae | Florenciella parvula | 98.7 |  |
| cDNA.97 | 890 | Florenciella parvula | 99.4 | Dictyochophyceae |  |  |  |
| cDNA.35 | 886 | AMT15_27B_4 | 97.6 | Dinoflagellate | Heterocapsa pygmaea | 97.2 |  |
| cDNA.10 | 893 | Phaeocystis cordata | 99.8 | Haptophyte |  |  |  |
| cDNA.15 | 837 | Phaeocystis cordata | 99.8 | Haptophyte |  |  |  |
| cDNA.19 | 883 | OLI16029 | 99.2 | Haptophyte | Chrysochromulina acantha | 98.7 |  |
| cDNA.60 | 894 | Syracosphaera pulchra | 99.1 | Haptophyte |  |  |  |
| cDNA.71 | 839 | PD6.17 | 98.4 | Haptophyte | Chrysochromulina sp. NIES-1333 | 98.3 |  |
| cDNA.81 | 873 | Chrysochromulina hirta | 95.8 | Haptophyte |  |  |  |
| cDNA.86 | 937 | Phaeocystis globosa | 100 | Haptophyte |  |  |  |
| cDNA.109 | 935 | Phaeocystis cordata | 99.2 | Haptophyte |  |  |  |
| cDNA.07 | 840 | FBB25 | 99.4 | MALV-I | Karlodinium micrum | 85.7 |  |
| cDNA.18 | 877 | FBB25 | 99.1 | MALV-I | Heterocapsa pygmaea | 87.1 |  |
| cDNA.57 | 919 | FBB25 | 98.1 | MALV-I | Karlodinium micrum | 87.2 |  |
| cDNA.73 | 836 | FBB25-Q2A12N5 | 99.3 | MALV-I | Karlodinium micrum | 86.0 |  |
| cDNA.76 | 916 | FBB25 | 99.4 | MALV-I | Karlodinium micrum | 86.9 |  |
| cDNA.87 | 919 | FBB25 | 99.2 | MALV-I | Karlodinium micrum | 86.5 |  |
| cDNA.110 | 922 | FBB25 | 99.3 | MALV-I | Karlodinium micrum | 86.8 |  |
| cDNA.04 | 865 | SCM37C36 | 91.1 | MALV-II | Amoebophrya sp. ex Gonyaulax | 90.3 |  |
| cDNA.49 | 909 | UEPACAPp3 | 99.8 | MALV-II | Amoebophrya sp. ex Ceratium | 92.6 |  |
| cDNA.41 | 838 | ME1-21 | 99.6 | MAST-1A | Developayella elegans | 90.8 |  |
| cDNA.03 | 884 | IND31.46 | 99.8 | MAST-1C | H.catenoides | 92.7 |  |
| cDNA.62 | 838 | FV18_3H10 | 100.0 | MAST-1C | H.catenoides | 93.0 |  |
| cDNA.09 | 925 | ME1-17 | 99.7 | MAST-2 | Pirsonia guinardiae | 90.2 |  |
| cDNA.36 | 919 | DH148-5-EKD53 | 99.0 | MAST-2 | Pirsonia guinardiae | 89.8 |  |
| cDNA.11 | 838 | SSRPD86 | 97.4 | MAST-3 | H.catenoides | 88.6 | BL010625.14 (100%) |
| cDNA.16 | 881 | SSRPD86 | 97.4 | MAST-3 | H.catenoides | 88.6 | BL010625.14 (99,2%) |
| cDNA.29 | 838 | SSRPD86 | 99.6 | MAST-3 | H.catenoides | 89.1 |  |
| cDNA.30 | 924 | SSRPD86 | 97.4 | MAST-3 | H.catenoides | 88.0 | BL010625.14 (100%) |
| cDNA.38 | 913 | UEPACOp5 | 99.4 | MAST-3 | Pirsonia punctigera | 87.7 | OLI11006 (94,7%) |
| cDNA.45 | 888 | NIF_3D5 | 95.4 | MAST-3 | Developayella elegans | 88.6 |  |
| cDNA.54 | 871 | SSRPD86 | 97.5 | MAST-3 | H.catenoides | 88.8 | BL010625.14 (100%) |
| cDNA.61 | 838 | SSRPD86 | 97.4 | MAST-3 | H.catenoides | 88.6 | BL010625.14 (100%) |
| cDNA.66 | 871 | SSRPD86 | 97.1 | MAST-3 | H.catenoides | 88.5 | BL010625.14 (99,9%) |
| cDNA.67 | 926 | SSRPD86 | 97.3 | MAST-3 | H.catenoides | 88.1 | BL010625.14 (100%) |
| cDNA.78 | 928 | SSRPD86 | 97.4 | MAST-3 | H.catenoides | 88.0 | BL010625.14 (99,9%) |
| cDNA.79 | 928 | SSRPD86 | 97.3 | MAST-3 | H.catenoides | 88.1 | BL010625.14 (99,9%) |
| cDNA.83 | 867 | MB10.48 | 98.1 | MAST-3 | Rhizidiomyces apophysatus | 91.6 | BL000921.38 (92,3%) |
| cDNA.84 | 925 | SSRPD86 | 97.5 | MAST-3 | H.catenoides | 88.1 | BL010625.14 (100%) |
| cDNA.88 | 925 | SSRPD86 | 97.5 | MAST-3 | H.catenoides | 88.1 | BL010625.14 (100%) |
| cDNA.90 | 925 | SSRPD86 | 97.4 | MAST-3 | H.catenoides | 88.0 | BL010625.14 (99,9%) |
| cDNA.94 | 865 | BL000921.38 | 92.0 | MAST-3 | Developayella elegans | 89.9 |  |
| cDNA.96 | 871 | SSRPD86 | 97.2 | MAST-3 | H.catenoides | 88.6 | BL010625.14 (99,7%) |
| cDNA.105 | 896 | SSRPD86 | 97.4 | MAST-3 | H.catenoides | 88.5 | BL010625.14 (100%) |
| cDNA.106 | 910 | ME1-28 | 99.4 | MAST-3 | H.catenoides | 88.0 | BL000921.18 (100%) |
| cDNA.107 | 911 | NIF_3D5 | 96.8 | MAST-3 | Developayella elegans | 88.0 |  |
| cDNA.111 | 879 | NIF_3D5 | 95.3 | MAST-3 | Developayella elegans | 88.3 |  |
| cDNA.17 | 877 | G03N10 | 100.0 | MAST-4 | Thraustochytriidae sp. HU1 | 88.2 |  |
| cDNA.27 | 917 | G03N10 | 99.8 | MAST-4 | Thraustochytriidae sp. HU1 | 87.9 |  |
| cDNA.44 | 893 | G03N10 | 100.0 | MAST-4 | Thraustochytriidae sp. HU1 | 88.0 |  |
| cDNA.77 | 896 | G03N10 | 99.7 | MAST-4 | Thraustochytriidae sp. HU1 | 87.6 |  |
| cDNA.80 | 939 | G03N10 | 99.6 | MAST-4 | Thraustochytriidae sp. Lsl2 | 87.9 | BL010625.50 (99,0%) |
| cDNA.82 | 935 | G03N10 | 99.7 | MAST-4 | Thraustochytriidae sp. Lsl2 | 88.0 |  |
| cDNA.85 | 945 | G03N10 | 99.8 | MAST-4 | Thraustochytriidae sp. Lsl2 | 88.0 |  |
| cDNA.95 | 946 | G03N10 | 99.8 | MAST-4 | Thraustochytriidae sp. Lsl2 | 88.0 |  |
| cDNA.98 | 917 | G03N10 | 99.9 | MAST-4 | Thraustochytriidae sp. HU1 | 87.9 |  |
| cDNA.99 | 940 | G03N10 | 99.3 | MAST-4 | Thraustochytriidae sp. Lsl2 | 87.7 |  |
| cDNA.100 | 937 | G03N10 | 100.0 | MAST-4 | Thraustochytriidae sp. Lsl2 | 99.1 |  |
| cDNA.14 | 906 | BL010320.6 | 97.8 | MAST-7 | Oblongichytrium sp. SEK 347 | 87.8 |  |
| cDNA.42 | 915 | BL010320.6 | 98.0 | MAST-7 | Oblongichytrium sp. SEK 347 | 87.9 |  |
| cDNA.53 | 915 | BL010320.6 | 97.9 | MAST-7 | Oblongichytrium sp. SEK 347 | 87.8 |  |
| cDNA.58 | 917 | BL000921.11 | 99.8 | MAST-7 | Rhizidiomyces apophysatus | 87.7 |  |
| cDNA.63 | 908 | BL010320.6 | 97.8 | MAST-7 | Oblongichytrium sp. SEK 347 | 87.7 |  |
| cDNA.23 | 864 | ANT12-26 | 94.8 | MAST-8 | Oblongichytrium sp. SEK 347 | 89.7 |  |
| cDNA.51 | 838 | ANT12-26 | 94.0 | MAST-8 | Oblongichytrium sp. SEK 347 | 88.3 |  |
| cDNA.08 | 848 | ME1-24 | 91.2 | MAST-Xa | Thraustochytriidae sp. HU1 | 92.7 |  |
| cDNA.13 | 839 | SCM27C54 | 99.9 | MAST-Xb | Oblongichytrium sp. SEK 347 | 89.4 |  |
| cDNA.92 | 952 | SCM27C54 | 99.8 | MAST-Xb | Thraustochytriidae sp. HU1 | 91.7 |  |
| cDNA.69 | 911 | ME1-24 | 91.0 | MAST-Xc | Thraustochytriidae sp. HU1 | 90.8 |  |
| cDNA.101 | 875 | Oblongichytrium sp. SEK 347 | 93.2 | MAST-Xc |  |  |  |
| cDNA.47 | 859 | SA1_2E5 | 85.2 | Novel-A | Ancyromonas sigmoides | 85.1 |  |
| cDNA.68 | 853 | Gungnir neglectum | 87.1 | Novel-B |  |  |  |
| cDNA.26 | 923 | Pelagomonas calceolata | 100.0 | Pelagophyte |  |  |  |
| cDNA.32 | 868 | Pelagomonas calceolata | 99.9 | Pelagophyte |  |  |  |
| cDNA.33 | 892 | Pelagomonas calceolata | 100.0 | Pelagophyte |  |  |  |
| cDNA.52 | 835 | Pelagomonas calceolata | 99.9 | Pelagophyte |  |  |  |
| cDNA.59 | 963 | Pelagomonas calceolata | 99.9 | Pelagophyte |  |  |  |
| cDNA.65 | 871 | Pelagomonas calceolata | 99.8 | Pelagophyte |  |  |  |
| cDNA.93 | 951 | Pelagomonas calceolata | 100.0 | Pelagophyte |  |  |  |
| cDNA.103 | 924 | Pelagomonas calceolata | 99.8 | Pelagophyte |  |  |  |
| cDNA.108 | 896 | Pelagomonas calceolata | 100.0 | Pelagophyte |  |  |  |
| cDNA.24 | 864 | FS01AA11_01Aug05_5m | 99.9 | Picobiliphyte | Lagenoeca sp. antarctica | 89.1 |  |
| cDNA.31 | 878 | NW617.02 | 98.4 | Picobiliphyte | Lagenoeca sp. antarctica | 92.6 |  |
| cDNA.37 | 913 | NW617.02 | 98.4 | Picobiliphyte | Cyllamyces aberensis | 91.6 |  |
| cDNA.55 | 884 | NW617.02 | 99.9 | Picobiliphyte | Neocallimastix frontalis | 92.3 |  |
| cDNA.72 | 837 | NW617.02 | 98.2 | Picobiliphyte | Lagenoeca sp. antarctica | 92.6 |  |
| cDNA.12 | 835 | BL000921.10 | 100.0 | Prasinophyte | Micromonas pusilla CCMP2099 | 98.4 |  |
| cDNA.20 | 837 | UEPAC34p4 | 99.5 | Prasinophyte | Micromonas pusilla | 99.4 |  |
| cDNA.56 | 905 | Micromonas pusilla, MBIC10095 | 100.0 | Prasinophyte |  |  |  |
| cDNA.102 | 945 | UEPAC34p4 | 99.6 | Prasinophyte | Micromonas pusilla | 99.5 |  |
| cDNA.104 | 868 | Crustomastix sp. MBIC10709 | 92.4 | Prasinophyte |  |  | PROSOPE.C5-25m.101 (97,4) |
| cDNA.02 | 876 | SSRPC45 | 90.0 | Radiolaria | Amphibelone anomala | 90.0 |  |
| cDNA.06 | 861 | SSRPC49 | 90.7 | Radiolaria | Acanthometra sp. | 89.7 |  |
| cDNA.89 | 850 | SSRPC45 | 91.1 | Radiolaria | Acanthometra sp. | 89.9 |  |
| cDNA.34 | 891 | NIF_1D10 | 95.1 | Stram (nov Phot)-A | Haramonas pauciplastida | 90.8 |  |
| cDNA.112 | 837 | NIF_1D10 | 95.5 | Stram (nov Phot)-A | Haramonas pauciplastida | 91.2 |  |
| cDNA.40 | 838 | Paraphysomonas imperforata VS1 | 92.1 | Stram (nov Phot)-B |  |  |  |
| cDNA.64 | 872 | BL000921.5 | 98.5 | Stram (nov Phot)-C | Polypodochrysis teissieri (Pingui) | 92.7 |  |
| cDNA.01 | 779 | NW414.42 | 99.7 | Telonema | Telonema antarcticum | 96.3 |  |
| cDNA.25 | 858 | RA001219.10 | 97.4 | Telonema | Telonema subtilis | 97.3 |  |
| cDNA.113 | 910 | PD6.20 | 98.5 | Telonema | Telonema antarcticum | 96.8 |  |
