# Supplementary material for: New Insights into the Diversity of Marine Picoeukaryotes
Source: PLoS One. 2009 Sep 29;4(9):e7143. doi: 10.1371/journal.pone.0007143 (PMC2747013; doi:10.1371/journal.pone.0007143)
Supplement: Table S3 — List of closest blast results for the DNA based clone library (0.16 MB DOC) [file pone.0007143.s004.doc]

| Name | read | Closest match | % | Phylogenetic group | Closest cultured match | % | Shorter seq |
| --- | --- | --- | --- | --- | --- | --- | --- |
| RFM1.11 | 839 | C3_E031 | 96.2 | Chrysophyte | P.foraminifera | 91.4 | BL000921.17 (99,3%) |
| RFM1.58 | 840 | C3_E031 | 96.1 | Chrysophyte | Paraphysomonas foraminifera | 92.1 | BL000921.17 (99,3%) |
| RFM1.44 | 821 | YS18Ec27 | 92.4 | Copepoda | Stenopontius sp. | 92.6 |  |
| RFM1.12 | 838 | Proteomonas sulcata | 98.7 | Cryptophyta |  |  |  |
| RFM1.08 | 838 | SCM16C28 | 99.6 | Dinoflagellate | Prorocentrum triestinum | 98.9 |  |
| RFM1.23 | 838 | SCM15C83 | 99.4 | Dinoflagellate | Prorocentrum micans | 98.3 |  |
| RFM1.55 | 790 | AMT15_15B_12 | 99.2 | Dinoflagellate | Adenoides eludens | 92.1 |  |
| RFM1.50 | 921 | N10E02 | 97.8 | Haptophyte | Chrysochromulina acantha | 97.3 |  |
| RFM1.51 | 858 | Prymnesium zebrinum | 99.3 | Haptophyte |  |  |  |
| RFM1.01 | 834 | SSRPD94 | 99.6 | MALV-I | Heterocapsa pygmaea | 87.1 |  |
| RFM1.02 | 838 | Q2A12N5 | 99.9 | MALV-I | Karlodinium micrum | 87.1 |  |
| RFM1.05 | 838 | SSRPB86 | 100.0 | MALV-I | Karlodinium micrum | 87.3 |  |
| RFM1.06 | 837 | SCM37C51 | 95.3 | MALV-I | Ichthyodinium sp. PL | 84.8 |  |
| RFM1.14 | 833 | E131 | 99.0 | MALV-I | Ichthyodinium sp. PL | 86.5 |  |
| RFM1.15 | 838 | E131 | 96.2 | MALV-I | Ichthyodinium sp. PL | 86.5 |  |
| RFM1.22 | 834 | SSRPB86 | 99.9 | MALV-I | Karlodinium micrum | 87.3 |  |
| RFM1.36 | 985 | 18BR9 | 97.5 | MALV-I | Amphidinium semilunatum | 88.4 |  |
| RFM1.37 | 862 | E131 | 99.1 | MALV-I | Duboscquella Hamana/2003 | 92.1 |  |
| RFM1.40 | 967 | SCM28C151 | 99.3 | MALV-I | Karlodinium micrum | 86.9 |  |
| RFM1.42 | 952 | SSRPD88 | 99.4 | MALV-I | Gyrodinium galatheanum | 89.6 |  |
| RFM1.59 | 923 | SCM37C51 | 96.2 | MALV-I | Duboscquella Hamana/2003 | 87.6 |  |
| RFM1.64 | 997 | SCM38C44 | 97.9 | MALV-I | Pfiesteria-like HR1SSeptA5 | 86.3 |  |
| RFM1.03 | 838 | H02N5 | 97.7 | MALV-II | Amoebophrya, Gonyaulax | 92.8 | ENVP21819.00110 |
| RFM1.04 | 836 | H02N5 | 97.7 | MALV-II | Amoebophrya, Gonyaulax | 92.8 | RFM1.3 |
| RFM1.13 | 837 | AP-picoclone20 | 95.3 | MALV-II | Amoebophrya, Gymnodinium | 92.6 |  |
| RFM1.16 | 837 | RA001219.57 | 98.9 | MALV-II | Amoebophrya, Gonyaulax | 91.2 | Pseudopfiesteria shumwayae |
| RFM1.28 | 886 | SSRPC86 | 95.4 | MALV-II | Amoebophrya, Gonyaulax | 92.2 |  |
| RFM1.33 | 947 | SSRPB75 | 99.9 | MALV-II | Amoebophrya, Alexandrium | 89.7 |  |
| RFM1.46 | 953 | SSRPC31 | 99.3 | MALV-II | Amoebophrya, Gonyaulax | 90.6 |  |
| RFM1.53 | 958 | SSRPC31 | 99.4 | MALV-II | Amoebophrya Gonyaulax | 91.0 |  |
| RFM1.60 | 946 | SSRPC86 | 95.7 | MALV-II | Amoebophrya Gonyaulax | 92.5 |  |
| RFM1.67 | 720 | MB01.3 | 98.1 | MALV-II |  |  |  |
| RFM1.72 | 847 | BL010320.29 | 92.3 | MALV-II | Pfiesteria piscicida | 88.8 |  |
| RFM1.63 | 848 | NIF_3D5 | 92.0 | MAST-3 | Developayella elegans | 89.9 |  |
| RFM1.32 | 880 | G03N10 | 100.0 | MAST-4 | Thraustochytriidae sp. Lsl2 | 89.8 |  |
| RFM1.38 | 824 | UEPACCp4 | 99.3 | MAST-4 | Thraustochytriidae sp. PW19 | 90.7 |  |
| RFM1.48 | 992 | SSRPD78 | 99.7 | MAST-4 | Oblongichytrium sp. SEK 347 | 90.7 |  |
| RFM1.73 | 857 | UEPACCp4 | 99.3 | MAST-4 | Thraustochytriidae sp. Lsl2 | 90.5 |  |
| RFM1.18 | 839 | Oikopleura sp. | 93.9 | Metazoa, Appendicularia |  |  |  |
| RFM1.65 | 966 | Oikopleura sp | 96.1 | Metazoa, Appendicularia |  |  |  |
| RFM1.25 | 880 | Bathycoccus prasinos | 99.9 | Prasinophyte |  |  |  |
| RFM1.07 | 840 | SSRPC42 | 98.9 | Radiolaria | Spongodiscus biconcavus | 90.9 | D.I-2 (RAD-III) |
| RFM1.09 | 845 | p14C4 | 99.9 | Radiolaria | Spongodiscus biconcavus | 90.3 | D.I-2 (RAD-III) |
| RFM1.10 | 840 | Dicranastrum furcatum | 89.6 | Radiolaria |  |  | ENVP36162.00093 |
| RFM1.17 | 845 | p14C4 | 99.9 | Radiolaria | Spongodiscus biconcavus | 90.3 | D.I-2 (RAD-III) |
| RFM1.19 | 841 | C1_E045 | 98.6 | Radiolaria | Spongodiscus biconcavus | 90.8 | D.I-2 (RAD-III) |
| RFM1.21 | 840 | p14C4 | 99.8 | Radiolaria | Spongodiscus biconcavus | 90.5 | D.I-2 (RAD-III) |
| RFM1.24 | 839 | SSRPC76 | 98.0 | Radiolaria | Spongodiscus biconcavus | 91.0 | D.I-2 (RAD-III) |
| RFM1.26 | 910 | T37C4 | 97.6 | Radiolaria | Triastrum aurivillii | 91.6 |  |
| RFM1.27 | 926 | C1_E045 | 98.8 | Radiolaria | Spongodiscus biconcavus | 90.7 |  |
| RFM1.29 | 952 | OLI011-75m.62 | 99.0 | Radiolaria | Spongaster tetras | 91.8 |  |
| RFM1.30 | 938 | C1_E045 | 99.0 | Radiolaria | Didymocyrtis tetrathalamus | 92.8 |  |
| RFM1.31 | 850 | E187 | 99.2 | Radiolaria | Larcopyle butschlii | 92.3 |  |
| RFM1.34 | 982 | OLI011-75m.2(5) | 96.8 | Radiolaria | Triastrum aurivillii | 89.6 |  |
| RFM1.39 | 945 | p14C4 | 99.5 | Radiolaria | Spongaster tetras | 92.4 |  |
| RFM1.41 | 939 | SSRPB22 | 99.6 | Radiolaria | Spongaster tetras | 92.0 |  |
| RFM1.43 | 860 | NW614.49 | 99.8 | Radiolaria | Chaunacanthid 217 | 90.0 |  |
| RFM1.45 | 948 | OLI011-75m.2(5) | 95.2 | Radiolaria | Dicranastrum furcatum | 90.0 |  |
| RFM1.52 | 918 | C1_E045 | 98.9 | Radiolaria | Didymocyrtis tetrathalamus | 92.3 |  |
| RFM1.54 | 944 | DH147-EKD17 | 98.1 | Radiolaria | Acanthometra sp. | 89.7 |  |
| RFM1.57 | 967 | C1_E045 | 98.7 | Radiolaria | Spongodiscus biconcavus | 90.6 |  |
| RFM1.61 | 931 | OLI011-75m.2(5) | 96.8 | Radiolaria | Dicranastrum furcatum | 89.5 |  |
| RFM1.62 | 881 | C1_E045 | 98.6 | Radiolaria | Didymocyrtis tetrathalamus | 92.0 |  |
| RFM1.66 | 890 | C1_E045 | 98.8 | Radiolaria | Spongodiscus biconcavus | 92.0 |  |
| RFM1.69 | 888 | OLI011-75m.2(5) | 95.5 | Radiolaria | Dicranastrum furcatum | 89.9 |  |
