# Supplementary material for: New Insights into the Diversity of Marine Picoeukaryotes
Source: PLoS One. 2009 Sep 29;4(9):e7143. doi: 10.1371/journal.pone.0007143 (PMC2747013; doi:10.1371/journal.pone.0007143)
Supplement: Table S4 — Closest blast hits on sequences retrieved from the GOS < 0.8µm dataset (0.10 MB DOC) [file pone.0007143.s005.doc]

| **Closest Match** | **Accession** | **%** | **Taxonomy** |
| --- | --- | --- | --- |
| Guinardia solstherfothii | AY485511 | 97.48% | Bacillariophyta |
| Uncultured marine eukaryote clone SIF_4B4 | EF527067 | 99.3% | Ciliophora |
| Strobilidium sp. clone Strob00ssu_1 | AF399124 | 99.6% | Ciliophora |
| Uncultured marine eukaryote clone UEPAC05Np2 | AY129035 | 99.29% | Ciliophora |
| Uncultured eukaryote isolate C1_E042 | AY046638 | 96.38% | Ciliophora |
| Strombidium sp. | AY143565 | 92.09% | Ciliophora |
| Uncultured marine eukaryote clone NIF_4C5 | EF526769 | 96.96% | Dinophyceae |
| Uncultured eukaryote clone SCM15C84 | AY664996 | 90.2% | Dinophyceae |
| Uncultured marine eukaryote clone NA1_3A7 | AB120001 | 99.44% | Dinophyceae |
| Uncultured eukaryote clone TWS121 | DQ344761 | 99.0% | Dinophyceae |
| Lepidodinium viride | DQ499645 | 98.26% | Dinophyceae |
| Gymnodinium galatheanum | AF172712 | 98.6% | Dinophyceae |
| Uncultured eukaryote clone AMT15_33B_16 | EU780624 | 99.53% | Dinophyceae |
| Uncultured eukaryote clone SCM15C6 | AY665087| | 91.14% | Euglenozoa |
| Uncultured eukaryote clone hotxp1f3 | EU500064 | 98.03% | Haptophyceae |
| Chrysochromulina simplex | AM491021 | 100.0% | Haptophyceae |
| eukaryote clone OLI11008 | AJ402350 | 97.13% | MAST 1 |
| Uncultured eukaryote clone dhot1e8 | EU499993 | 100.00% | MAST 1 |
| Uncultured eukaryote clone SSRPD78 | EF172962 | 99.84% | MAST 4 |
| Uncultured marine eukaryote clone NIF_1D10 | EF526882 | 90.32% | MAST 8 |
| Uncultured eukaryote clone SCM15C10 | AY664959 | 100.00% | MALV-I |
| Uncultured eukaryote clone SCM28C139 | AY665031 | 99.71% | MALV-I |
| Uncultured marine eukaryote clone UEPACAPp5 | AY129054 | 94.66% | MALV-II |
| Uncultured marine picoplankton AP-picoclone15 | DQ386751 | 92.52% | MALV-II |
| eukaryote clone OLI11023 | AJ402335 | 97.0% | MALV-II |
| Amoebophrya sp. | AF472553 | 94.80% | MALV-II |
| Uncultured eukaryote clone AMT15_1B_36 | EU780604 | 99.6% | MALV-II |
| Amoebophrya sp. | AF472553 | 94.78% | MALV-II |
| Uncultured eukaryote clone AMT15_1B_36 | EU780604 | 99.6% | MALV-II |
| Uncultured marine eukaryote clone cLA12H06 | EU446389 | 96.74% | MALV-II |
| Amoebophrya sp. | AY208893 | 88.15% | MALV-II |
| Uncultured marine eukaryote clone UEPAC43p4 | AY129050 | 91.64% | MALV-II |
| Uncultured eukaryote clone SSRPD74 | EF172958 | 99.87% | MALV-II |
| Uncultured eukaryote clone SSRPD74 | EF172958 | 99.88% | MALV-II |
| Uncultured marine eukaryote clone CD8S2 | DQ647524 | 99.76% | MALV-II |
| Blastodinium navicula | DQ317538 | 80.56% | Novel Alveolates X |
| Uncultured eukaryote isolate E230 | AY443014 | 82.81% | Novel Alveolates X |
| Collozoum inerme | AY266295 | 97.4% | Polycystinea |
| Collozoum inerme | AY266295 | 97.2% | Polycystinea |
| Collozoum inerme | AY266295 | 96.8% | Polycystinea |
| Collozoum inerme | AY266295 | 96.00% | Polycystinea |
| Collozoum inerme | AY266295 | 98.1% | Polycystinea |
| Siphonosphaera cyathina | AF091145 | 84.0% | Polycystinea |
| Collozoum inerme | AY266295 | 94.8% | Polycystinea |
| Collozoum inerme | AY266295 | 97.21% | Polycystinea |
| Collozoum inerme | AY266295 | 98.68% | Polycystinea |
| Collozoum inerme | AY266295 | 97.07% | Polycystinea |
| Thalassophysa pelagica | AY266296 | 98.73% | Polycystinea |
| Collozoum pelagicum | AF091146 | 92.34% | Polycystinea |
| Collozoum inerme | AY266295 | 93.33% | Polycystinea |
| Collozoum inerme | AY266295 | 95.6% | Polycystinea |
| Collozoum inerme | AY266295 | 95.71% | Polycystinea |
| Uncultured eukaryote clone SCM27C26 | AY665072 | 98.7% | Polycystinea |
| Sphaerozoum punctatum | AF018161 | 96.26% | Polycystinea |
| Thalassophysa pelagica | AY266296 | 93.90% | Polycystinea |
| Collozoum inerme | AY266295 | 95.19% | Polycystinea |
| Rhaphidozoum acuferum | AF091147 | 94.51% | Polycystinea |
| Ostreococcus lucimarinus | CP000592 | 100.00% | Prasinophyceae |
| Ostreococcus sp. RCC 143 | AY425310 | 99.79% | Prasinophyceae |
| Ostreococcus lucimarinus | CP000592 | 99.2% | Prasinophyceae |
| Uncultured prasinophyte clone BL0009210 | AY425318 | 100.00% | Prasinophyceae |
| Uncultured prasinophyte clone BL0009210 | AY425318 | 100.00% | Prasinophyceae |
| Ostreococcus lucimarinus | CP000592 | 99.1% | Prasinophyceae |
| Ostreococcus lucimarinus | CP000592 | 99.64% | Prasinophyceae |
| Bathycoccus prasinos | AY425315 | 99.31% | Prasinophyceae |
| Uncultured marine eukaryote clone UEPACDp3 | AY425315 | 100.00% | Prasinophyceae |
| Ostreococcus lucimarinus | CP000592 | 100.0% | Prasinophyceae |
| Ostreococcus lucimarinus | CP000592 | 100.00% | Prasinophyceae |
| Uncultured taxopodid-like clone LC22_5EP_23 | DQ504355 | 99.3% | Taxopodida |
