# Supplementary material for: New Insights into the Diversity of Marine Picoeukaryotes
Source: PLoS One. 2009 Sep 29;4(9):e7143. doi: 10.1371/journal.pone.0007143 (PMC2747013; doi:10.1371/journal.pone.0007143)
Supplement: Table S5 — Closest blast hits on sequences retrieved from the GOS 0.8 - 3 µm dataset (0.08 MB DOC) [file pone.0007143.s006.doc]

| **Closest Match** | **Accession** | **%** | **Taxonomy** |
| --- | --- | --- | --- |
| Uncultured marine eukaryote clone UEPACAHp3 | DQ369016 | 91.96% | Cercozoa |
| Acanthocoepsis unguiculata | L10823 | 92.32% | Choanoflagellida |
| Uncultured eukaryote isolate P6X4-3 | AJ579337 | 93.02% | Chrysophyceae |
| Uncultured eukaryote clone SSRPE02 | EF172998 | 100.00% | Chrysophyceae |
| Uncultured eukaryote clone SSRPD79 | EF172963 | 99.24% | Cryptophyta |
| Uncultured eukaryote clone AMT15_33B_9 | EU780620 | 99.87% | Dinophyceae |
| Chrysochromulina simplex | AM491021 | 98.7% | Haptophyceae |
| Prymnesium nemamethecum | AM491004 | 91.83% | Haptophyceae |
| Chrysochromulina rotalis | AM491025 | 100.00% | Haptophyceae |
| Unidentified prymnesiophyte clone OLI16010 | AF107081 | 97.55% | Haptophyceae |
| Chrysochromulina ericina | AM491030 | 100.00% | Haptophyceae |
| eukaryote clone OLI11056 | AJ402351 | 99.53% | Haptophyceae |
| eukaryote clone OLI11008 | AJ402350 | 99.33% | MAST 1 |
| eukaryote clone OLI11008 | AJ402350 | 99.82% | MAST 1 |
| eukaryote clone OLI11008 | AJ402350 | 99.86% | MAST 1 |
| Uncultured eukaryote clone dhot1e8 | AJ402350 | 100.00% | MAST 1 |
| Uncultured eukaryote clone BL000921.38 | AY381200 | 93.5% | MAST 3 |
| Uncultured marine eukaryote clone NIF_3D5 | EF526815 | 95.51% | MAST 3 |
| Uncultured marine eukaryote clone UEPACRp5 | AY129069 | 99.73% | MAST 3 |
| eukaryote clone OLI11150 | AJ402355 | 99.28% | MAST 7 |
| eukaryote clone OLI11150 | AJ402355 | 99.30% | MAST 7 |
| Rhizidiomyces apophysatus | AF163295 | 91.2% | MASTX |
| Uncultured eukaryote clone hotxp1d6 | AF163295 | 95.66% | MASTX |
| Uncultured marine eukaryote clone SA2_1E2 | EF527126 | 100.0% | MALV-I |
| Uncultured eukaryote clone SCM28C139 | AY665031 | 98.0% | MALV-I |
| Uncultured marine eukaryote clone UEPACCp1 | AY129029 | 99.26% | MALV-I |
| Uncultured marine eukaryote clone SA2_1E2 | EF527126 | 98.94% | MALV-I |
| Uncultured eukaryote clone SCM37C30 | AY665056 | 97.20% | MALV-II |
| Uncultured marine eukaryote clone UEPACBp4 | AY129045 | 88.36% | MALV-II |
| Uncultured eukaryote clone AMT15_15B_12 | EU780615 | 98.84% | MALV-II |
| Uncultured marine eukaryote clone UEPACAFp5 | AY129057 | 99.60% | MALV-II |
| Uncultured marine eukaryote clone UEPACAPp3 | AY129041 | 98.8% | MALV-II |
| Uncultured marine eukaryote clone UEPAC41p3 | AY129042 | 99.78% | MALV-II |
| Uncultured marine eukaryote clone NS51B233 | AJ829830 | 92.91% | MALV-II |
| Uncultured eukaryote clone SSRPD91 | EF172992 | 99.02% | MALV-II |
| Uncultured marine eukaryote clone UEPACFp3 | AY129040 | 96.46% | MALV-II |
| Uncultured eukaryote clone Q2A12N10 | EF172973 | 100.00% | MALV-II |
| Amoebophrya sp. | AF472553 | 96.79% | MALV-II |
| Uncultured marine eukaryote clone UEPAC42p4 | AY129049 | 94.11% | MALV-II |
| Uncultured eukaryote clone Q2A12N10 | EF172973 | 100.00% | MALV-II |
| Amoebophrya sp. | AF472553 | 89.56% | MALV-II |
| eukaryote clone OLI11005 | AJ402349 | 98.33% | MALV-II |
| Pelagomonas calceolata | EF455763 | 99.86% | Pelagophyceae |
| Uncultured marine eukaryote clone AD6S.10 | DQ647540 | 99.40% | Picobiliphytes |
| Uncultured phototrophic eukaryote clone RA000907.54 | DQ222877 | 99.12% | Picobiliphytes |
| Uncultured marine eukaryote clone UEPACWp1 | AF525854 | 100.00% | Prasinophyceae |
| Ostreococcus sp. RCC 393 | AY425311 | 100.00% | Prasinophyceae |
